# Targeting PGK1: A New Frontier in Breast Cancer Therapy Under Hypoxic Conditions

**DOI:** 10.3390/cimb46110725

**Published:** 2024-10-30

**Authors:** Jiayong Cui, Shengjun Chai, Rui Liu, Guoshuang Shen

**Affiliations:** 1 Clinical Medicine College, Graduate School of Qinghai University, Xining 810000, China; ys221002100823@qhu.edu.cn (J.C.); ys221002100819@qhu.edu.cn (S.C.); ys241002141035@qhu.edu.cn (R.L.); 2 Breast Disease Diagnosis and Treatment Cencer, Affiliated Hospital of Qinghai University, Xining 810000, China

**Keywords:** breast cancer, hypoxia, HIF-1α, glycolysis, PGK1

## Abstract

Breast cancer represents one of the most prevalent malignant neoplasms affecting women, and its pathogenesis has garnered significant scholarly interest. Research indicates that the progression of breast cancer is intricately regulated by glucose metabolism. Under hypoxic conditions within the tumor microenvironment, breast cancer cells generate ATP and essential biosynthetic precursors for growth via the glycolytic pathway. Notably, phosphoglycerate kinase 1 (PGK1) is intimately associated with the regulation of hypoxia-inducible factors in breast cancer and plays a crucial role in modulating glycolytic processes. Further investigation into the role of PGK1 in breast cancer pathogenesis is anticipated to identify novel therapeutic targets and strategies. This review consolidates current research on the regulation of glucose metabolism and the function of PGK1 in breast cancer within hypoxic conditions. It aims to offer a significant theoretical foundation for elucidating the mechanisms underlying breast cancer progression and metastasis, thereby facilitating the development of innovative treatment approaches.

## 1. Introduction

Breast cancer is one of the most common malignant tumors in women and poses a serious threat to women’s health [1]. Although the diagnosis and treatment of breast cancer have made some progress, it is still the main cause of cancer-related death in women around the world [2,3], so it is necessary to further explore the occurrence and development mechanism of breast cancer at a deeper level.

In recent years, the advancement of breast cancer towards a malignant state has been thought to be closely related to the regulation of glucose metabolism in a hypoxic environment [4,5]. A study found that when cells are in a low-oxygen environment, their glucose metabolism pathways are significantly altered, and these changes involve the regulation of several key metabolic genes and signaling pathways. In particular, the expression and activity of some genes encoding glycolytic enzymes and lactate dehydrogenase may be significantly enhanced in breast cancer cells, thus promoting the survival, proliferation, and invasion ability of tumor cells [6]. In addition, regulation of glucose metabolism in breast cancer cells under a hypoxic environment also involves the participation of various transcription factors and signal transduction pathways, such as the hypoxia-inducible factor (HIF)-1α pathway [7,8]. These factors can not only directly affect the expression of glycometabolic enzymes but also indirectly affect the metabolic adaptability of breast cancer cells in a low-oxygen environment by regulating the level of transcription and translation.

To sum up, in-depth understanding of the regulatory mechanism of glucose metabolism in breast cancer cells under a hypoxic environment has important clinical significance for the development of new targeted therapeutic strategies. Therefore, this review will summarize the research progress of glucose metabolism and its related genes in breast cancer under a hypoxic environment, and explore their future application prospects in breast cancer.

## 2. Breast Cancer

At present, breast cancer has surpassed lung cancer to become the most common malignant tumor with the highest incidence in women worldwide, and it is also the leading cause of tumor-related death in women [9,10]. According to cancer data for 2024, the estimated number of cases of breast cancer in women accounts for 32% of all cancers in women. Although the five-year survival rate for breast cancer patients has improved as testing methods and treatment strategies continue to improve, the incidence of breast cancer in women has increased slowly at a rate of about 0.6% per year since 2000. As a result, breast cancer remains one of the major health challenges facing women worldwide [11].

Surgery is the main treatment for breast cancer [12], but medication also plays an important role [13]. However, the emergence of molecular typing of breast cancer has led to the development of drugs to treat breast cancer from common chemotherapy to targeted therapy, endocrine therapy, and immunotherapy [14,15]. Genetic testing is crucial in precision medicine for breast cancer, focusing on genes such as BRCA1 and BRCA2 to detect mutations that may raise breast cancer risk [16]. Additionally, advances in molecular biology of breast cancer have revealed the existence of distinct molecular subtypes. Breast cancer can be broadly classified into three primary subtypes based on molecular phenotype [17,18]: the hormone receptor (HR)-positive subtype encompasses tumors that express estrogen receptors (ERs) and/or progesterone receptors (PRs), and these tumors typically exhibit sensitivity to endocrine therapy [19]. HR+ breast cancer mainly includes luminal A and luminal B subtypes, which differ in biology, prognosis, and treatment response. Luminal A is usually ER+, PR+, human epidermal growth factor receptor 2 (HER2)-negative, with low proliferation, leading to a better prognosis and good response to endocrine therapy, often without the need for chemotherapy or only low-intensity chemotherapy [20]. Luminal B is typically ER+ and may be PR+/-, with HER2 status varying. It has a high proliferation index, often resulting in a poorer prognosis and requiring more aggressive treatments such as endocrine therapy and chemotherapy, especially if HER2-positive. The HER2-positive subtype is distinguished by the amplification of the HER2 gene and is frequently associated with ERs and PRs. Tumors of this subtype may exhibit a favorable response to HER2-targeted therapies, such as Trastuzumab. Conversely, triple-negative breast cancer (TNBC) is characterized by a lack of ERs, PRs, and HER2, and is generally associated with a more aggressive clinical course and resistance to standard hormonal therapies and HER2-targeted interventions [21]. 

The prevailing approach in contemporary medical practice involves formulating a patient’s treatment plan predicated on the molecular classification of breast cancer; however, traditional subtyping has limitations because it ignores interactions with the tumor microenvironment (TME) [22]. Metastasis is a principal determinant of mortality in breast cancer [23]. Research indicates that luminal A breast cancers are relatively less aggressive, while luminal B breast cancers exhibit greater aggressiveness; HER2-positive breast cancers demonstrate a moderate level of aggressiveness, whereas TNBC is identified as the most aggressive subtype [24]. Tumor aggressiveness is intricately associated with malignancy, characterized by local invasion, distant metastasis, tissue destruction, cellular heterogeneity, and immune evasion [25]. At the cellular level, the success of metastatic dissemination hinges on the tumor cell capacity for adaptation, which includes metabolic reprogramming to navigate the hostile TME and secure essential nutrients to satisfy the energy requirements for cellular proliferation [26]. Metabolic reprogramming has been identified as a pivotal characteristic of cancer, with modifications in glucose metabolism being especially significant. Cancer cells, irrespective of aerobic or hypoxic (anaerobic) conditions, frequently exhibit a preference for glucose metabolism via glycolysis, culminating in the production of lactic acid [27]. Despite substantial progress in breast cancer research over recent decades, numerous challenges persist that necessitate further comprehensive investigation. It is imperative to explore the mechanisms underlying breast cancer occurrence and progression, as well as to identify effective biomarkers to enhance diagnosis, treatment, and prognostic evaluation. The reprogramming of glucose metabolism plays a crucial role in tumor development and progression. Consequently, research into the mechanisms of glucose metabolism in breast cancer continues to be a critical area of study.

## 3. Tumor Microenvironment

**Figure 1:**
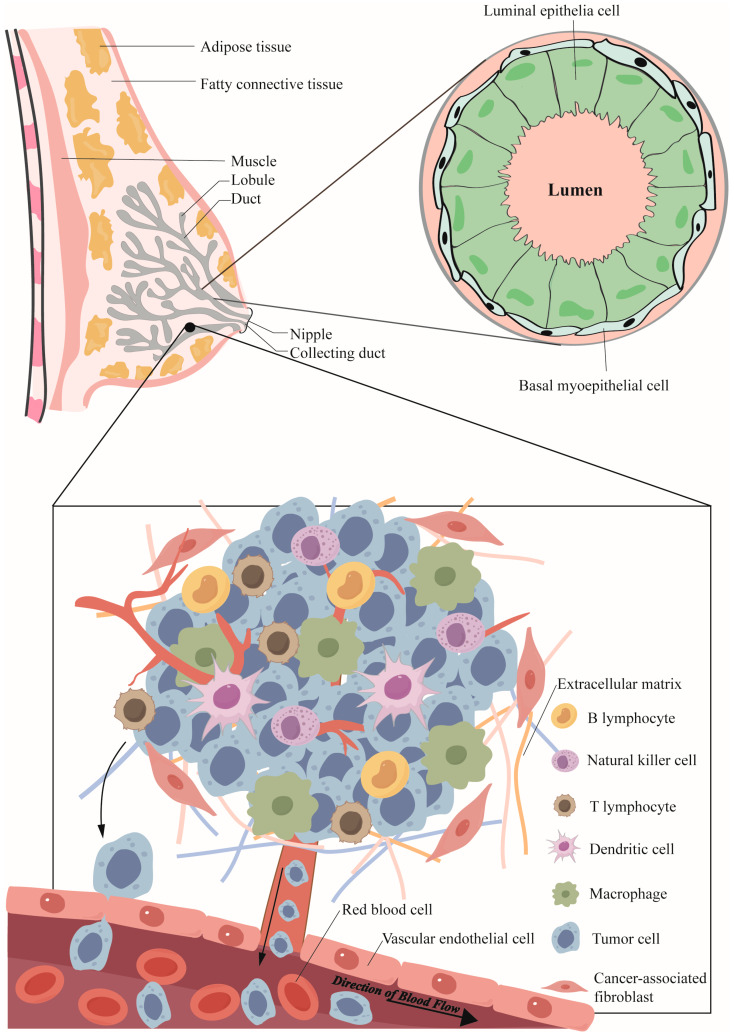
Schematic representation of the tumor microenvironment in breast cancer and its cellular composition. This figure details the two main mechanisms by which tumor cells invade the vascular system. The first mechanism, intravascular infiltration, illustrates tumor cells entering the arterial lumen through gaps between endothelial cells. The second mechanism shows tumor cells bypassing the endothelial layer and directly entering through connected vessels. Arrows indicate the direction of tumor cell movement.

TME constitutes an intricate network comprising diverse cellular and non-cellular components surrounding the tumor cells (Figure 1). Predominant cell types within this microenvironment include tumor-associated macrophages, lymphocytes, fibroblasts, and endothelial cells, which modulate tumor progression through the secretion of growth factors, cytokines, and chemokines [28]. These elements, encompassing processes such as angiogenesis [29], immune cell infiltration [30], and extracellular matrix remodeling [31], collectively facilitate tumor cell proliferation, invasion, and metastasis [32]. 

The hypoxic environment constitutes a critical characteristic of the TME, exerting a substantial influence on tumor progression [33,34]. This form of hypoxia arises primarily due to two interrelated factors. Firstly, the rapid proliferation of tumor cells escalates oxygen demand within tumor tissues, surpassing the oxygen-supply capacity of the existing vasculature, thereby inducing tissue hypoxia [35,36]. Secondly, the irregular angiogenesis and rapid expansion of tumor tissue impede effective blood circulation, further hindering the efficient delivery of oxygen to tumor cells [37]. Moreover, the aberrant vascular architecture of the tumor, coupled with the incomplete integrity of the vascular wall, can further aggravate the pathophysiology [29]. The proliferation of tumor-associated stromal cells and fibroblasts additionally impairs oxygen delivery and diffusion, thereby contributing to the prevalence of hypoxic conditions within the TME [38,39]. 

A hypoxic environment significantly influences the growth, metastasis, and drug resistance of tumor cells [40]. Under hypoxic conditions, tumor cells enhance their proliferative and metastatic capabilities by activating signaling pathways that adapt to the low-oxygen environment [34]. Under hypoxic conditions, tumor cells adapt to environmental stress and facilitate cell proliferation and metastasis through the activation of various HIF signaling pathways, including HIF-1α and HIF-2α. These factors regulate cellular metabolic pathways, enhance glycolysis and lactic acid production, and improve the adaptability and aggressiveness of tumor cells by modulating mechanisms of apoptosis inhibition and promoting angiogenesis [41]. Furthermore, hypoxic environments can induce increased chemotherapy resistance in tumor cells by augmenting DNA damage-repair mechanisms and mitigating oxidative stress responses [42]. This acquired resistance not only complicates treatment protocols but also diminishes patient survival rates and overall treatment efficacy.

Therefore, a comprehensive investigation into the relationship between the TME, hypoxia-inducible factors, and breast cancer is crucial for elucidating the pathogenesis of the disease. Such research may also yield valuable insights for the development of novel therapeutic targets and personalized treatment strategies. Findings in this area are anticipated to significantly advance the diagnosis and treatment of breast cancer, enhance therapeutic outcomes, and prolong patient survival.

## 4. Glucose Metabolism

Glucose metabolism constitutes a fundamental cellular process essential for maintaining normal cellular function and growth. Under typical physiological conditions, cells metabolize glucose to produce energy via glycolysis and oxidative phosphorylation pathways. Glycolysis, a critical component of glucose metabolism, involves the enzymatic breakdown of glucose into pyruvate, yielding energy and various metabolites. Subsequently, under aerobic conditions, cells further convert glucose-derived pyruvate into energy through the oxidative phosphorylation pathway. In certain malignant tumor cells, this process is inhibited, leading to energy production via lactic acid fermentation, a phenomenon known as the Warburg effect [43]. This effect describes the preference of cancer cells to utilize glycolysis over mitochondrial oxidative phosphorylation for glucose metabolism, even in the presence of sufficient oxygen, during proliferation and metastasis [44]. Although glycolysis generates less energy compared to aerobic oxidative phosphorylation, it occurs at a faster rate, thereby supporting the rapid proliferation characteristic of tumor cells [45]. In tumor cells, alterations in glucose metabolism induced by the TME result in a preferential utilization of the glycolytic pathway for energy production. Such a metabolic shift not only supplies the necessary energy for tumor cells but also generates amino acids, lipids, and other essential substances required for cellular growth. Consequently, this metabolic reprogramming facilitates tumor proliferation and metastasis. The metabolic pathway not only facilitates the rapid proliferation and dissemination of tumor cells but also enhances their aggressiveness and resistance to pharmacological treatments [46]. The Warburg effect has emerged as a significant marker of cancer and is intimately associated with carcinogenesis [47]. In the context of breast cancer, the activation of the glycolytic pathway is closely linked to tumor initiation and progression, thereby establishing it as a critical target for therapeutic intervention. 

Current research has extensively investigated the potential of inhibitors targeting the glycolytic pathway and key enzymes involved in glucose metabolism for the treatment of breast cancer. Several studies have demonstrated that disrupting the glycolytic pathway or inhibiting crucial enzymes such as phosphofructokinase-1 and phosphofructokinase-2 can effectively impede the proliferation and metastasis of cancer cells [48,49,50,51,52,53]. Furthermore, inhibitors targeting other significant enzymes, including hexokinase and layered double hydroxides, have also exhibited promising efficacy against cancer [54,55,56,57]. Additionally, modulating the glucose metabolism of cancer cells has the potential to enhance the efficacy of radiotherapy and chemotherapy while mitigating associated side effects [58,59]. However, these treatments continue to encounter several challenges. Firstly, certain inhibitors may induce side effects in normal cells, leading to adverse reactions [60]. Secondly, breast cancer cells may circumvent the inhibition of the glycolytic pathway by activating alternative metabolic pathways, thereby diminishing the efficacy of the inhibitors [61,62]. Consequently, it is imperative to conduct further research into the mechanisms of the glycolysis pathway in breast cancer and to develop more effective treatment strategies. In conclusion, additional research on glycolysis and the optimization of related approaches are crucial for enhancing the efficacy of cancer treatment. 

Building upon an in-depth investigation into the role of glucose metabolism in breast cancer, researchers have begun to explore the glucose metabolism pathway as a novel therapeutic strategy for breast cancer treatment. Utilizing the cancer genome atlas (TCGA) database for bioinformatics analysis, Jiang et al. identified seven glycolysis-related genes significantly associated with overall survival in patients [63]. The genes listed in Table 1, which include phosphoglycerate kinase 1 (PGK1), syndecan 1 (SDC1, also known as CD138), syndecan 3 (SDC3), nucleoporin 43 (NUP43), interleukin 13 receptor subunit alpha 1 (IL13RA1), adenylate kinase 3 (AK3), and calcium voltage-gated channel subunit alpha 1H (CACNA1H), play a crucial role in the etiology of breast cancer. Previous research has indicated that PGK1, SDC1, SDC3, NUP43, and IL13RA1 may contribute to the development and progression of breast cancer [64,65,66,67,68,69,70]. These factors potentially influence tumor growth and metastatic capacity through various mechanisms, thereby facilitating the advancement of breast cancer. Conversely, AK3 and CACNA1H are suggested to exert inhibitory effects on breast cancer progression [71,72]. The investigation of these genes and their corresponding proteins not only deepens our understanding of the molecular mechanisms underlying breast cancer advancement but also provides essential insights for the development of novel therapeutic strategies and targeted pharmacological interventions.

The role of glucose metabolism in breast cancer represents a complex and significant area of research. A comprehensive understanding of the mechanisms underlying glucose metabolism in the onset and progression of breast cancer is crucial for elucidating the pathophysiological processes involved. This knowledge may inform the development of novel therapeutic strategies and interventions. 

## 5. Phosphoglycerate Kinase 1

**Figure 2:**
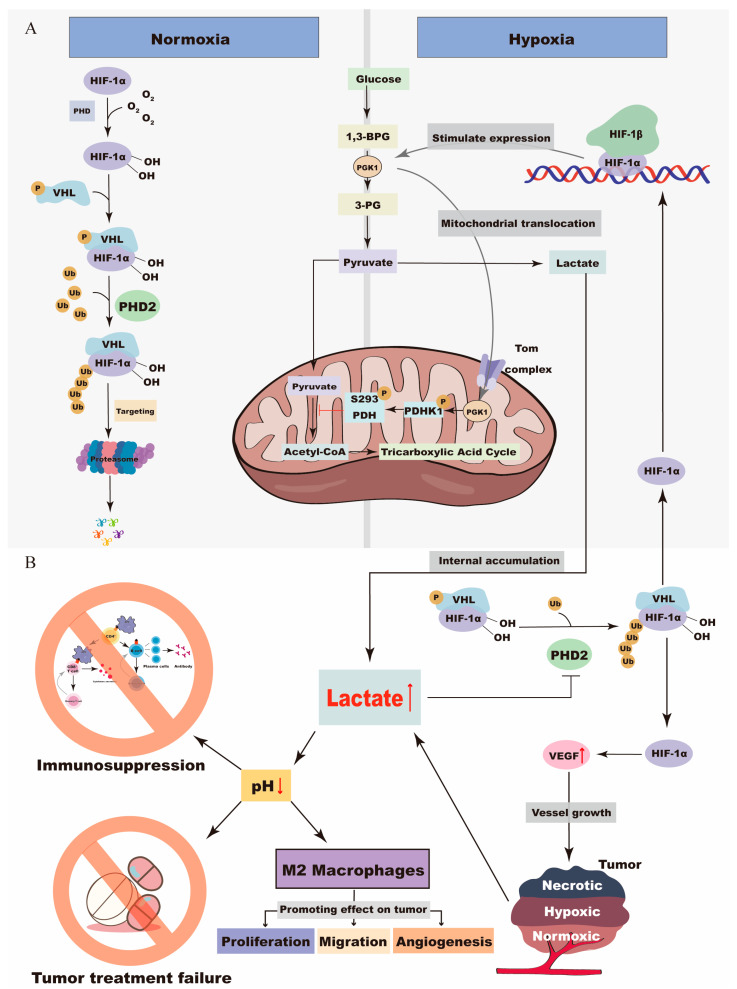
Interaction between HIF-1α and PGK1 in the Warburg Effect. (**A**). Under hypoxic conditions in the TME, HIF-1α accumulation leads to increased PGK1 expression, enhancing glycolysis and pyruvate production while inhibiting the TCA cycle and prompting lactic acid accumulation. (**B**). The resulting lactic acid buildup lowers pH, promoting cancer progression and inhibiting HIF-1α ubiquitination, which further increases PGK1 expression, thereby sustaining a feedback loop that exacerbates tumor growth.

PGK1 functions as a rate-limiting enzyme integral to the glycolytic pathway, facilitating the conversion of 1,3-bisphosphoglycerate and ADP to 3-phosphoglycerate and ATP [65,73]. This reaction represents the initial ATP-generating step in glycolysis, a critical process for energy production in most living cells. In the context of solid tumors, particularly under hypoxic conditions within the TME, PGK1-mediated glycolysis supplies essential energy to tumor cells, thereby playing a significant role in the progression of various cancers [74]. Under normoxic conditions, HIF-1α binds to the von Hippel–Lindau (VHL) tumor suppressor protein, leading to its ubiquitination and subsequent rapid degradation by prolyl hydroxylase domain-containing protein 2 (PHD2) [75]. Conversely, under hypoxic conditions within the TME, HIF-1α evades degradation [76], allowing it to dimerize with HIF-1β and activate the expression of PGK1 [77]. This upregulation of PGK1 enhances the glycolytic pathway [73]. With the facilitation of the translocase of the outer membrane (TOM) complex, PGK1 undergoes mitochondrial translocation and subsequently phosphorylates threonine 338 on pyruvate dehydrogenase kinase 1 (T338PDHK1) within the mitochondria [78]. This event results in the phosphorylation of serine on pyruvate dehydrogenase (S293PDH), thereby inhibiting the conversion of pyruvate to acetyl-CoA and ultimately suppressing the tricarboxylic acid (TCA) cycle [79]. The mitochondrial translocation of PGK1 results in the inhibition of pyruvate oxidation within the mitochondria and an increased conversion of pyruvate to lactate in the cytoplasm (Figure 2) [80]. Previous studies have corroborated this process by demonstrating that defects in the recombinant expression of PGK1 T378P mutants lead to either mitochondrial translocation of PGK1 or kinase inactivation in PGK1-depleted cells. This was achieved through CRISPR/Cas9-mediated knockdown of PGK1S203A, which subsequently inhibited the hypoxia and epidermal growth factor receptor (EGFR) activation-induced reduction in lactic acid production and mitochondrial pyruvate oxidation. Further in vivo functional experiments demonstrated that substituting endogenous PGK1 with a mitochondrial translocation-deficient mutant, PGK1 S203A, inhibited cancer cell proliferation and induced apoptosis [81].

In the hypoxic TME, there is an accumulation of HIF-1α, which subsequently upregulates the expression of PGK1. The augmented levels of PGK1 boost glycolytic activity, leading to increased production of pyruvate. Simultaneously, PGK1 facilitates a shift toward mitochondrial reliance while suppressing the tricarboxylic acid (TCA) cycle, culminating in the accumulation of lactic acid. This buildup of lactic acid not only lowers the pH, promoting the progression of cancer but also inhibits the ubiquitination of HIF-1. The resulting elevated levels of HIF-1 further enhance PGK1 expression, thus perpetuating a self-sustaining cycle that exacerbates cancerous activity.

However, the elevated production of lactic acid poses significant challenges for cancer patients (Figure 2). Elevated levels of lactic acid can directly precipitate the decline and apoptosis of immune cells, including natural killer cells, dendritic cells, and CD8+ T cells, potentially leading to adverse outcomes such as immunosuppression and the failure of tumor chemotherapy [82,83,84,85]. Furthermore, an excessive accumulation of lactic acid can lower the pH of the extracellular environment, which may promote the polarization of M2 macrophages, subsequently, this facilitates the proliferation, migration, and angiogenesis of tumor cells [86]. Moreover, lactic acid inhibits PHD2, preventing normal degradation of HIF-1α [87]. Consequently, HIF-1α binds to HIF-1β, thereby enhancing the expression of PGK1 and establishing a positive feedback loop. Additionally, HIF-1α facilitates the synthesis of vascular endothelial growth factor (VEGF), which in turn stimulates tumor angiogenesis and consequently accelerates cancer progression [88,89].

A substantial upregulation of PGK1 expression has been observed across various breast cancer subtypes, with the expression levels of PGK1 demonstrating a strong correlation with tumor malignancy and prognosis [90,91]. Furthermore, PGK1 has a potential utility as a diagnostic biomarker for breast cancer and is anticipated to serve as a critical indicator for early screening and prognostic evaluation [92,93,94]. A multitude of studies have corroborated the association between PGK1 overexpression and the pathogenesis and progression of breast cancer, highlighting its role in facilitating tumor growth and metastasis. Research indicates that downregulation of PGK1 expression can inhibit proliferation, migration, and invasion of breast cancer cells, as well as reverse epithelial–mesenchymal transition (EMT) [95,96,97]. In a study conducted by Sun et al., breast cancer patients undergoing Paclitaxel chemotherapy were analyzed, revealing that those with elevated PGK1 expression exhibited shorter overall survival compared to patients with lower PGK1 levels. This finding suggests that high PGK1 expression may be a significant factor contributing to poor prognosis in breast cancer patients treated with Paclitaxel [65]. Subsequent investigations into PGK1 revealed that the viability of breast cancer cell lines with downregulated PGK1 expression was markedly diminished following Paclitaxel treatment. Furthermore, it was observed that the downregulation of PGK1 led to the activation of apoptotic proteins, specifically cleaved caspase-3 and Bax, while concurrently inhibiting the expression of the anti-apoptotic protein Bcl-2 in Paclitaxel-treated TNBC cell lines [98]. Therefore, the downregulation of PGK1 expression may be associated with apoptosis-mediated sensitivity to Paclitaxel in TNBC. Research indicates that the overall expression level of PGK1 is significantly higher in HER-2-positive breast cancer compared to HER-2 negative breast cancer. Furthermore, treatment with the specific inhibitor Herceptin in HER-2-positive breast cancer has been shown to markedly reduce PGK1 protein expression, thereby effectively inhibiting the proliferation of breast cancer cells. This suggests that downregulating the expression of PGK1 could be pivotal in the treatment of breast cancer [99]. Consequently, investigating PGK1 may represent a significant avenue for future research in the diagnosis and treatment of this disease.

**Figure 3:**
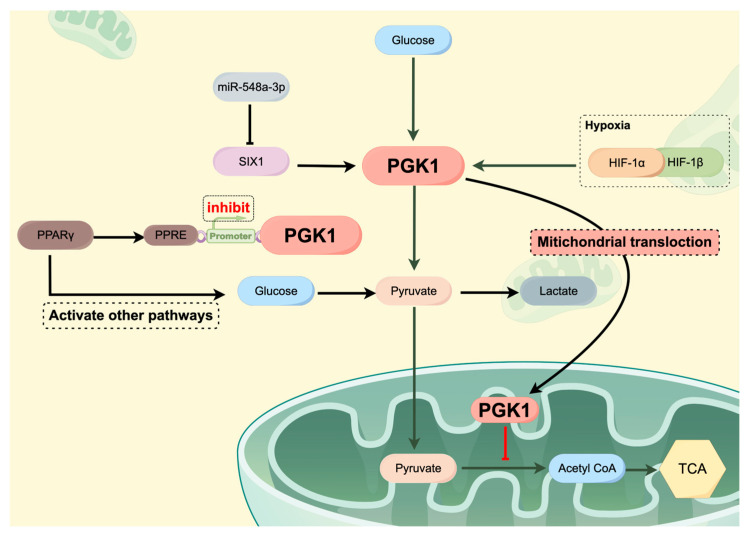
The Role of PGK1 in Breast Cancer Metabolism and Signaling Pathways. This schematic illustrates the pivotal role of PGK1 in modulating metabolic pathways within the context of breast cancer. It delineates PGK1’s interactions across several key signaling axes, including HIF-1α, MYC, and SIX1/HBO1, which are instrumental in regulating cellular glycolysis and the Warburg effect.

Furthermore, research into the mechanisms underlying breast cancer has identified PGK1 as a crucial component in various signaling pathways (Figure 3). Specifically, HIF-1α upregulates a series of glycolytic genes during anaerobic glycolysis, with PGK1 serving as a downstream target. Within the HIF-1α/PGK1 signaling pathway, PGK1 and HIF-1α establish a positive feedback loop. Activation of this pathway has been demonstrated to facilitate the process of EMT, thereby enhancing the metastatic potential of breast cancer and promoting the Warburg effect [77]. Furthermore, activation of the MYC/PGK1 pathway has been shown to amplify the Warburg effect in breast cancer, which in turn contributes to the initiation and progression of the disease [100]. Recent domestic studies have revealed that the function of PGK1 is modulated by non-coding RNA. In breast cancer, the transcription factor SIX1 induces the transcription of PGK1 by binding to its promoter region and recruiting the histone acetyltransferase HBO1, thereby promoting the Warburg effect and tumor growth both in vitro and in vivo. However, SIX1 is directly inhibited by miR-548a-3p, leading to decreased PGK1 expression and reduced aerobic glycolysis. In other words, miR-548a-3p downregulates PGK1 to inhibit glycolysis [101]. Concurrently, PPARγ can inhibit PGK1 gene transcriptional activity and induce glycolysis by targeting the common peroxisome proliferator-activated receptor response element (PPRE) hormone response element within the PGK1 gene promoter region [102]. These seemingly contradictory phenomena may be related to factors such as the diversity of metabolic pathways, environmental dependency effects, indirect activation of glycolysis, and temporal dynamics. To fully understand the impact of PGK1 on metabolic pathways, further research is needed to elucidate these mechanisms and verify their effectiveness under specific conditions. This is crucial for revealing the role of PGK1 in metabolic diseases and cancer, as well as its potential in therapeutic applications.

Despite advancements in the investigation of PGK1 in breast cancer, numerous critical issues remain to be addressed. These include elucidating the upstream regulatory mechanisms governing PGK1 in the pathogenesis and progression of breast cancer, as well as understanding the interactions between PGK1 and other signaling pathways. In their investigation of the regulatory relationship between basic helix-loop-helix family member e41 (BHLHE41) and HIF-1α in osteosarcoma, Hu et al. observed a downregulation in the mRNA expression level of the HIF-1α target gene PGK1 upon knockdown of BHLHE41. Notably, they also identified a positive correlation between BHLHE41 expression and poor prognosis in osteosarcoma patients [103]. This finding contrasts with the results of several studies on BHLHE41 in breast cancer. Montagner et al. demonstrated through in vitro studies that BHLHE41 is capable of inhibiting HIF-mediated cancer cell migration in TNBC [104]. Their subsequent in vivo experiments corroborated these findings, revealing that BHLHE41 effectively impedes HIF-induced invasion and metastasis. Beyond its impact on breast cancer cells, BHLHE41 may also play a role in breast cancer progression by modulating processes such as immune cell activity and angiogenesis within the TME [105]. Research conducted by Liu, Zhang and their colleagues has demonstrated that BHLHE41 plays a regulatory role in the activity of tumor-associated immune cells, thereby influencing the host immune system’s ability to recognize and eliminate tumors [106,107]. Additionally, the study by Liao et al. revealed that BHLHE41 may impact tumor growth and metastasis by modulating the process of angiogenesis, which in turn affects the blood supply and nutrient availability to the tumors [108]. Therefore, BHLHE41 is recognized as a tumor suppressor gene in breast cancer. Currently, the potential impact of alterations in BHLHE41 expression levels on PGK1 expression and subsequent breast cancer progression remains an area that necessitates further investigation and validation.

The research advancements concerning PGK1 in breast cancer offer significant insights into the pathogenesis and therapeutic strategies for this malignancy. Currently, additional investigations are imperative to elucidate the precise mechanisms by which PGK1 influences the onset and progression of breast cancer. Such studies are essential to furnish a robust scientific foundation for the early diagnosis and effective treatment of the disease. It is anticipated that future research will further delineate the role of PGK1 in breast cancer, thereby providing a more comprehensive theoretical basis for its treatment and prevention.

## 6. Summary

Surgical intervention continues to be the primary modality in the treatment of breast cancer; however, alternative therapeutic approaches are increasingly being explored, owing to advancements in medical research [109,110,111]. These alternatives, which encompass targeted therapy, immunotherapy, radiotherapy, and combination therapies, have demonstrated promising outcomes, albeit with notable challenge. Despite the progress in non-surgical treatments for breast cancer, these modalities are not devoid of limitations. The heterogeneity of breast cancer and the emergence of resistance to therapies persist as substantial obstacles [22,112]. Nevertheless, continuous research and clinical trials persist in refining these methodologies, thereby providing optimism for the development of more effective and individualized treatment options in the future.

In hypoxic conditions, cells typically enhance the activity of the glycolytic pathway to sustain survival and proliferation. Glycolysis, a crucial metabolic pathway, generates energy by converting glucose into pyruvate and lactic acid. In the context of breast cancer, the glycolytic pathway under hypoxic conditions is recognized as a significant mechanism for maintaining the survival and proliferation of cancer cells. Research has demonstrated that the activation of the glycolytic pathway is closely associated with the malignancy and prognosis of breast cancer [113]. PGK1, a crucial enzyme in the glycolytic pathway, plays a significant role in the conversion of phosphorylated glucose. Recent research has demonstrated that PGK1 expression levels are frequently elevated in breast cancer and are closely associated with the invasive and metastatic capabilities of cancer cells [114]. Furthermore, PGK1 can be detected in the peripheral blood of patients, suggesting its potential as a highly promising biomarker for breast cancer screening. However, the precise role of PGK1 in the pathogenesis of breast cancer remains inadequately understood. Given its potential as a target for innovative approaches in early diagnosis and treatment, further investigation into PGK1 is warranted.

In conclusion, the advancements in research on the glycolytic pathway and the glycolysis-related gene PGK1 in the context of breast cancer under hypoxic conditions offer valuable insights into the mechanisms and therapeutic targets of metabolic reprogramming in breast cancer. Future investigations should delve deeper into the glycolytic pathway and the intricate regulatory mechanisms of PGK1 in breast cancer progression, with the aim of providing a more robust scientific foundation for personalized treatment and prognosis evaluation in breast cancer. As research on PGK1 advances, it is anticipated that our comprehension of breast cancer pathogenesis will be enhanced, thereby facilitating the development of more efficacious treatment modalities for patients. Future investigations should aim to elucidate the intricate molecular mechanisms underlying these regulatory pathways and examine their potential clinical applications. Such efforts are essential for improving the prognosis and quality of life for individuals affected by breast cancer.

## Figures and Tables

**Table 1:** Seven genes related to glycolysis have been found to be significantly associated with overall survival in patients with breast cancer.

Gene	NCBI Gene ID	Malignant Behaviors in Breast Cancer	References (PMID)
PGK1	5230	Proliferation, invasion, EMT, metastasis, immune escape, and therapeutic resistance	39192221, 25867275 [64,65]
SDC1	6382	Proliferation, invasion, EMT, metastasis, and therapeutic resistance	37069585, 37056938 [66,67]
SDC3	9672	Positively associated with tumor lymph node metastases, higher clinical stage, and HER-2 positivity	23351331 [68]
NUP43	348995	DNA amplification	29402145 [69]
IL13RA1	3597	Proliferation, metastasis	28634667 [70]
AK3	50808	Tumor suppressor function	32196830 [71]
CACNA1H	8912	Targeted growth inhibition of brain metastases	31129098 [72]

## Abbreviations

AK3	adenylate kinase 3
BHLHE41	basic helix-loop-helix family member e41
CACNA1H	calcium voltage-gated channel subunit alpha 1H
EMT	epithelial–mesenchymal transition
ER	estrogen receptors
EGFR	epidermal growth factor receptor
HER2	human epidermal growth factor receptor 2
HIF	hypoxia-inducible factor
HR	hormone receptor
IL13RA1	interleukin 13 receptor subunit alpha 1
NUP43	nucleoporin 43
PHD2	prolyl hydroxylase domain-containing protein 2
PGK1	phosphoglycerate kinase 1
PPRE	peroxisome proliferator-activated receptor response element
PR	progesterone receptors
SDC1	syndecan 1
SDC3	syndecan 3
S293PDH	serine 293 on pyruvate dehydrogenase
TCA	tricarboxylic acid
TCGA	the cancer genome atlas
TME	tumor microenvironment
TNBC	triple-negative breast cancer
TOM	translocase of the outer membrane
T338PDHK1	threonine 338 on pyruvate dehydrogenase kinase 1
VEGF	vascular endothelial growth factor
VHL	von Hippel–Lindau
